# Phylogenetic Distribution, Ultrastructure, and Function of Bacterial Flagellar Sheaths

**DOI:** 10.3390/biom10030363

**Published:** 2020-02-27

**Authors:** Joshua Chu, Jun Liu, Timothy R. Hoover

**Affiliations:** 1 Department of Microbiology, Cornell University, Ithaca, NY 14853, USA; jc2568@cornell.edu; 2 Microbial Sciences Institute, Department of Microbial Pathogenesis, Yale University, West Haven, CT 06516, USA; jliu@yale.edu; 3 Department of Microbiology, University of Georgia, Athens, GA 30602, USA

**Keywords:** flagellum, flagellar sheath, *Helicobacter*, *Vibrio*, cardiolipin

## Abstract

A number of Gram-negative bacteria have a membrane surrounding their flagella, referred to as the flagellar sheath, which is continuous with the outer membrane. The flagellar sheath was initially described in *Vibrio metschnikovii* in the early 1950s as an extension of the outer cell wall layer that completely surrounded the flagellar filament. Subsequent studies identified other bacteria that possess flagellar sheaths, most of which are restricted to a few genera of the phylum Proteobacteria. Biochemical analysis of the flagellar sheaths from a few bacterial species revealed the presence of lipopolysaccharide, phospholipids, and outer membrane proteins in the sheath. Some proteins localize preferentially to the flagellar sheath, indicating mechanisms exist for protein partitioning to the sheath. Recent cryo-electron tomography studies have yielded high resolution images of the flagellar sheath and other structures closely associated with the sheath, which has generated insights and new hypotheses for how the flagellar sheath is synthesized. Various functions have been proposed for the flagellar sheath, including preventing disassociation of the flagellin subunits in the presence of gastric acid, avoiding activation of the host innate immune response by flagellin, activating the host immune response, adherence to host cells, and protecting the bacterium from bacteriophages.

## 1. Introduction

The bacterial flagellum is a complex organelle used for motility and is organized into three basic structures referred to as the basal body, hook and filament. Of these structures, the filament is the most prominent, forming a thin, helical structure that is typically 5–10 µm in length and is several times longer than the body of the bacterial cell. The filament is composed of tens of thousands of copies of a single flagellin protein or of multiple closely related flagellin proteins that self-assemble to form a hollow, tubular structure. In most bacterial species, the flagellar filament is exposed directly to the surrounding medium. The filament in several genera of Gram-negative bacteria, however, is surrounded by a membranous sheath that is contiguous with the outer membrane. Flagella in these bacteria are located almost exclusively at the cell pole, and occur as a single flagellum at one cell pole (polar flagellum), as a single flagellum at each cell pole (amphitrichous or bipolar flagella) or as multiple flagella at one cell pole (lophotrichous flagella). In bacteria with a lophotrichous arrangement of flagella, each flagellum is enclosed within a separate sheath. Other types of flagellar sheaths have been described, such as one found in the marine magnetotactic bacterium MO-1, which has a flagellar sheath composed of glycoprotein [1]. The MO-1 flagellar sheath differs further from membranous flagellar sheaths in that it surrounds a flagellar bundle consisting of multiple flagella rather than surrounding each flagellum [1]. Spirochetes enclose their flagella within the periplasmic space, which is somewhat analogous to the flagellar sheath in that the flagella of spirochetes are separated from the surrounding medium by a membrane. For additional information on the periplasmic flagella of spirochetes, we refer the reader to a recent review by Wolgemuth [2]. For the purposes of our review, we focus on bacteria that possess membranous flagellar sheaths. It has been nearly four decades since the last comprehensive review of the bacterial flagellar sheath [3], which was a major impetus for this review.

## 2. Phylogenetic Distribution of Flagellar Sheaths

Accurately assessing how widely distributed flagellar sheaths are among bacterial species is not a trivial task since reports on novel species often fail to indicate the presence or absence of a flagellar sheath. Moreover, when reports of novel bacterial species do indicate the presence of a flagellar sheath, they often omit a description of the ultrastructure of the sheath or do not include electron micrographs that clearly show the ultrastructure of the sheath. With these caveats in mind, membranous flagellar sheaths are found primarily in a handful of genera that are scattered throughout the phylum Proteobacteria. Within the Proteobacteria, representatives from five classes (Alphaproteobacteria, Betaproteobacteria, Gammaproteobacteria, Deltaproteobacteria and Epsilonproteobacteria) are reported to possess a membranous flagellar sheath. In addition to the Proteobacteria, some members of the phylum Planctomyces appear to have membranous flagellar sheaths, including *Pirella marina* and several members of the genus *Planctomycetes* [4,5].

Members of Alphaproteobacteria that have flagellar sheaths include *Seliberia stellate, Azospirillum brasilense*, *Rhodospirillum centenum*, and *Brucella melitensis*, all of which possess a single polar flagellum [6,7,8,9]. Within the Betaproteobacteria, the soil bacterium and plant pathogen *Robbsia andropogonis* (formerly known as *Burkholderia andropogonis*, *Pseudomonas andropogonis*, *Pseudomonas stizolobii*, as well as other names) has a single polar sheathed flagellum [10]. Busse and Auling indicate that members of the genus *Achromobacter*, which belong to the class Betaproteobacteria, have a peritrichous arrangement of sheathed flagella [11]. They do not report whether the *Achromobacter* flagellar sheaths are membranous, although the ultrastructure of *Achromobacter xylosoxidans* flagella appears consistent with that of a membranous flagellar sheath [12]. If the flagella of *Achromobacter* are indeed surrounded by a membranous sheath, this would be the only example of a bacterium with peritrichous sheathed flagella of which we are aware. Members of Gammaproteobacteria that have flagellar sheaths include *Halorhodospira adbelmalekii* [13], several species of *Pseudoalteromonas* [14,15,16,17], and most or all *Vibrio* species [18]. All of these members of Gammaproteobacteria possess either a single or multiple polar flagella that they use for swimming. In addition to producing a polar sheathed flagellum for swimming, various marine *Vibrio* species, including *Vibrio parahaemolyticus*, *Vibrio alginolyticus*, *Vibrio harveyi*, and *Vibrio shilonii*, elaborate lateral flagella that are used for rapid movement on surfaces [19,20,21]. The lateral flagella of the marine *Vibrio* species, as well as those of *A. brasilense* and *R. centenum*, lack a sheath [8,19,20,21,22], indicating that sheath biosynthesis is not inherently linked with flagellum biogenesis in these bacteria. *Bdellovibrio bacteriovorus*, *Bacteriovorax stolpii*, and *Bacteriovorax starrii*, which are predators of other Gram-negative bacteria, are members of the class Deltaproteobacteria that possess a single polar sheathed flagellum [23,24,25]. Within the class Epsilonproteobacteria, only members of the genus *Helicobacter* are reported to have flagellar sheaths. Most *Helicobacter* species possess a single sheathed polar flagellum or bipolar sheathed flagella; although the most extensively studied species, *Helicobacter pylori*, has lophotrichous sheathed flagella, and several *Helicobacter* species have unsheathed polar flagella. Interestingly, *Helicobacter* species that possess flagellar sheaths and *Helicobacter* species that have unsheathed flagella appear to segregate into distinct phylogenetic groups [26].

## 3. Composition of Flagellar Sheaths

**Figure 1:**
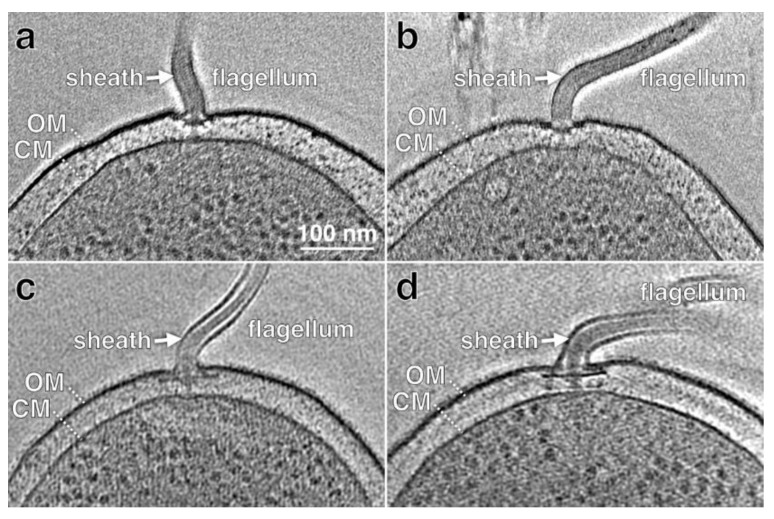
Cryot-ET reconstructions of intact cells show sheathed flagella. (**a**,**b**) Two representative sections from cryo-ET reconstructions of *V. cholerae* cells. (**c**,**d**) Two representative sections from cryo-ET reconstructions of *H. pylori* cells. The arrows indicate the flagellar sheath. For each flagellum, note the central core that consists of the hook and filament. The outer (OM) and cytoplasmic membranes (CM) are indicated.

Early ultrastructural studies of flagellar sheaths from various bacteria revealed two electron dense layers separated by a region of less electron density, consistent with the flagellar sheath being a unit membrane [3]. For some bacteria, including *B. bacteriovorus*, *B. melitensis*, *H. pylori*, and *V. fischeri*, a bulb-like structure is typically observed at the distal end of the flagellar sheath [7,27,28,29]. The detailed electron micrographs from the early studies suggested the flagellar sheath was contiguous with the outer membrane, and recent cryo-electron tomography (cryo-ET) studies of sheathed flagella from various bacteria have yielded detailed images that confirm the structural continuity between the outer membrane and flagellar sheath [30,31,32,33,34]. Figure 1 shows tomograms of *V. cholerae* and *H. pylori* sheathed flagella (authors’ data).

Early studies on the flagellar sheath sought to determine if the composition of the sheath was similar to that of the outer membrane by using antibodies directed against lipopolysaccharide (LPS) or other surface antigens in the outer membrane. Hranitzky and co-workers reported for *V. cholerae* that antibodies directed against a crude flagellar sheath preparation reacted strongly with a component in both the sheath and surface of the cell body [35]. Conversely, Yang and co-workers found that antibodies directed against a purified antigen from the cell body of *V. cholerae* cross-reacted with and immobilized the flagellum [36]. Although Hranitzky and co-workers reported that antibodies directed against *V. cholerae* LPS did not bind to the flagellar sheath [35], a subsequent study with *V. cholerae* found that anti-LPS did indeed recognize the flagellar sheath [37]. While these studies indicated that at least some components are shared between the outer membrane and flagellar sheath, determining whether specific macromolecules localize to the flagellar sheath required further biochemical characterization of isolated flagellar sheaths.

Little information is available on the lipid composition of the flagellar sheath for any bacterium, but the data that are available are intriguing. Thomashow and Rittenberg reported that the LPS of the flagellar sheath of *B. bacteriovorous* was moderately enriched (~2.7-fold) for the fatty acid nonadecenoic acid (C_19:1_) and depleted greatly (~17-fold) in β-hydroxymyristic acid (3-OH C_14:0_) compared to LPS from bdellovibrios grown on *Escherichia coli* as a host [29]. This observation indicates *B. bacteriovorous* partitions specific LPS species into the flagellar sheath that differ from those in the outer membrane. Interestingly, the total LPS (i.e., LPS from both the outer membrane and flagellar sheath) from bdellovibrios grown axenically (i.e., on medium instead of host cells) was similar to the flagellar sheath LPS in that it was enriched for nonadecenoic acid and depleted for β-hydroxymyristic acid [29]. Using immunogold labelling, Norqvist and Wolf-Watz identified a surface antigen in the fish pathogen *Vibrio anguillarum* that localized specifically to the flagellar sheath [38]. The *V. anguillarum* surface antigen was resistant to proteinase K, but sensitive to periodic acid treatment. In addition, the antigen was absent in mutants that had transposon insertions in *virA* and *virB*, which encode enzymes involved in LPS O1 antigen biosynthesis. Collectively, these findings indicate the *V. anguillarum* surface antigen is LPS [38], and further suggest that like *B. bacteriovorus*, *V. anguillarum* partitions specific LPS species to the flagellar sheath. The mechanisms these bacteria use to segregate specific LPS species to the flagellar sheath are unknown.

In an examination of the fatty acid composition of *H. pylori* flagellar sheaths, Geis and co-workers found there were no fatty acids uniquely associated with the flagellar sheath, but the fatty acid composition profile of the sheath does differ from that of the whole cell membranes [39]. Cardiolipin is a phospholipid that accumulates in regions of membranes that have negative curvature, such as the cell pole and septal regions in rod-shaped bacteria [40,41,42,43]. Given the propensity of cardiolipin to accumulate in membranes with negative curvature, one might expect the flagellar sheath to contain significant amounts of cardiolipin. Consistent with this hypothesis, the *H. pylori* flagellar sheath appears to contain high amounts of cardiolipin [44]. In further support of the hypothesis, the two most abundant fatty acids in *H. pylori* flagellar sheaths, myristic acid (C_14:0_) and cyclopropane nonadecanoic acid (C_19:0_ cyc) [39], are also the two most common fatty acids in cardiolipin species from *H. pylori* [45,46,47].

A number of studies have investigated the proteins associated with bacterial flagellar sheaths, although the information on flagellar sheath proteins is still scant. Knowledge of the proteins that localize to the flagellar sheath is critical for understanding the function of the sheath. Early studies on characterizing flagellar sheath proteins relied on serological approaches or SDS-polyacrylamide gel electrophoresis to identify proteins that appeared to be enriched in the flagellar sheath [29,35,39,48,49,50,51]. These studies typically reported the sizes of the putative flagellar sheath proteins, but did not identify or further characterize the proteins. The *B. bacteriovorus* flagellar sheath was reported to have substantially less total protein (23%–28% dry weight) than that of outer membranes from other bacteria, which typically ranges from 40 to 70% [29]. Using monoclonal antibodies to an outer membrane fraction from *H. pylori* NCTC 11637, Doig and Trust identified six protein antigens that either localized within or were associated with the outer membrane, but did not recognize the flagellar sheath, which suggested the proteomes of the outer membrane and flagellar sheath of *H. pylori* differ from each other [48]. Bari and co-workers identified three outer membrane proteins associated with the *V. cholerae* flagellum-OmpU and OmpT, which are porins, and VC1894, which is a predicted collagen-binding surface adhesion [52]. Disrupting *ompU* or *ompT* in *V. cholerae* resulted in several flagellum-associated defects, including reduced motility, thinner flagella, increased proportion of non-flagellated cells, and increased release of flagellin into the growth medium [52]. These findings suggest OmpU and OmpT help to stabilize the *V. cholerae* flagellar sheath.

An important question is whether there are proteins that localize preferentially to the flagellar sheath. One protein reported to localize specifically to the *H. pylori* flagellar sheath is the *H. pylori* adhesion A (HpaA), although there are conflicting reports on the localization of this protein. HpaA was first described as a hemagglutinin that was shown by immunogold labelling to be located on the cell surface, but was not detectable on the flagellar sheath [53]. A subsequent study indicated HpaA occurred predominantly in the cytoplasmic fraction of Sarkosyl-solubilized cells, with only trace amounts of HpaA in the inner membrane fraction and no detectable HpaA in the outer membrane fraction [54]. Other immunogold labelling studies showed HpaA was specifically localized to the flagellar sheath [51,55]; while a later immunogold labeling study examined localization of HpaA in five *H. pylori* strains and detected HpaA on both the flagellar sheath and the bacterial surface in all of the strains [56]. The case for HpaA being a flagellar sheath protein is very compelling, and some of the discrepancies between the reports on the surface location of the protein may be attributed to differences in growth phases of the *H. pylori* cultures, media used for growing the bacteria, strain variability or choice of antibody [55,56]. 

In a study of *H. pylori* genes predicted to encode proteins secreted by the type V (autotransporter) pathway, Radin and co-workers found one of these proteins localized specifically to the flagellar sheath, which was determined by immunogold electron microscopy and fluorescence microscopy using an antibody to a c-myc tag introduced within the autotransporter [57]. Given the association of the autotransporter with the flagellar sheath, the researchers designated the protein as FaaA (flagellar-associated autotransporter A). Disrupting *faaA* resulted in reduced motility and a variety of defects in flagellum biosynthesis or stability, which included increased number of non-flagellated cells, reduced number of flagella per cell, increased frequency of broken flagella, and increased proportion of flagella that localized to nonpolar sites [57]. *faaA* is not part of any of the known flagellar gene regulons in *H. pylori* [58]. FaaA is synthesized and localized to the cell surface in a *H. pylori* 26695 strain that does not produce flagella [59]. Taken together, these findings suggest a role for FaaA in assembly of the flagellum and/or flagellar sheath, but expression of *faaA* is not tightly coupled with the expression of flagellar genes.

The physiological role of FaaA is not known, although it is required for optimal colonization in a mouse animal model by *H. pylori* during early stages of infection [57]. The colonization deficiency of the *faaA* mutant may result from the defects in motility and flagellum biosynthesis since *H. pylori* must be motile to penetrate the gastric mucus layer to colonize the gastric mucosa [60]. Proteins secreted by the autotransporter pathway have two domains, a secreted passenger domain and a β-barrel domain that inserts in the outer membrane and facilitates transport of the passenger domain across the outer membrane [61]. Passenger domains participate in a variety of cellular functions, including enzymatic activities (e.g., proteases, lipases/esterases), contact-dependent growth inhibition, immune evasion, cytotoxicity, cyto-/hemolysis, adherence, biofilm formation, auto-agglutination, and activation of actin polymerases for intracellular motility [61]. Depending on the autotransporter, the passenger domain is cleaved and released outside the cell after it is transported across the outer membrane or it remains linked to the β-barrel domain and is exposed on the cell surface. Since the c-myc tag used to examine the localization of FaaA was introduced into the passenger domain [57], FaaA belongs to this later class of autotransporters.

## 4. Rotation of the Sheathed Flagellum

Researchers who initially studied flagellar sheaths raised questions about how to apply the rotary model for the bacterial flagellum mechanism. Fuerst proposed two models for how the flagellar filament and sheath cooperated in motility [62]. The first model proposed the filament and sheath rotate together. This model requires the flagellar sheath to be rigid and the intersection of the base of the flagellar sheath and outer membrane to be discontinuous and fluid to enable the sheath to rotate with the filament. The sheath would also interact with the filament, perhaps through hydrophobic interactions, to generate a rigid membrane structure. In the second model, which makes fewer assumptions about the nature of the outer membrane and is seemingly more plausible, the filament rotates freely within a flexible wave-propagating sheath [62]. In this second model, the membrane of the flagellar sheath must be flexible enough to allow distortion by the rotational forces induced by the filament, but robust enough to remain associated with the cell body. Fuerst proposed experiments to examine the movement of polystyrene latex beads attached to the flagellar sheath through anti-sheath antibodies as a possible way to distinguish between his two models [62]; however, to the best of our knowledge, there are no reports that address the behavior of the flagellar sheath as the filament rotates.

Rotation of the sheathed flagellum of various *Vibrio* species is a major source for outer membrane vesicles (OMVs) that are released from the bacterial cell [63,64]. Aschtgen and co-workers demonstrated that the amount of OMVs released is proportional to the number of sheathed flagella per cell [63]. Specifically, the researchers showed that *V. cholerae*, which has a single polar sheathed flagellum, released fewer OMVs than *V. parahaemolyticus* or *V. fischeri*, which have multiple polar sheathed flagella. The researchers also showed that *E. coli*, which has unsheathed peritrichous flagella, released the fewest amount of OMVs, and a non-flagellated *E. coli* strain released the same amount of OMVs as its parental strain [63]. It is not known how flagellar rotation results in release of OMVs, but it seems likely that they are shed from the flagellar sheath as the flagellum rotates. Membrane blebs have been observed at the tip and shaft of *Vibrio* flagellar sheaths [65,66], and these blebs may be the source of OMVs that are released during rotation of the flagellum [63].

The outer membrane of Gram-negative bacteria is an asymmetrical lipid bilayer with LPS at the outer leaflet and phospholipids at the inner leaflet, and serves as an effective barrier to antibiotics, detergents and other toxic compounds. Exposure of bacterial cells to antimicrobial peptides or metal chelating agents such as EDTA leads to shedding of LPS and allows phospholipids from the inner leaflet to move into the outer leaflet, thereby compromising the outer membrane as a barrier [67]. Loss of LPS and other macromolecules associated with the flagellar sheath as the flagellum rotates could similarly lead to the migration of phospholipids from the inner leaflet to the outer leaflet, which could be deleterious to the bacterium. Consistent with this hypothesis, the *B. bacteriovorus* flagellar sheath was reported to have a higher proportion of phospholipids than typical outer membranes, and the authors of this study speculated that this might account for the unusual sensitivity of bdellovibrios to detergents [29]. Gram-negative bacteria have mechanisms for removing phospholipids from the outer leaflet of the outer member to maintain lipid asymmetry [68,69]. If shedding of sheath material does indeed compromise the flagellar sheath as a barrier, one might expect some bacteria that possess flagellar sheaths to have robust mechanisms for maintaining the lipid asymmetry of the outer membrane and sheath.

## 5. Biogenesis of the Flagellar Sheath

Little is known regarding the biosynthesis of the flagellar sheath in any bacterial species, which makes any attempt to attribute the phylogenetic distribution of the flagellar sheath to horizontal gene transfer or convergent evolution highly speculative. Regardless of the evolutionary history of the flagellar sheath, it likely is not coincidental that almost all bacteria reported to have a flagellar sheath possess polar flagella. The bacterial cell pole has unique physiochemical properties that may have facilitated the evolution of a membranous sheath at this location. For example, the accumulation of cardiolipin at the bacterial cell pole is attributed to its ability to form clusters or microdomains, which exhibit a high intrinsic curvature and therefore have a lower energy when localized to regions of the membrane with negative curvature [70,71]. In addition to forming microdomains, cardiolipin induces other changes in the physical properties of membranes that may be critical for assembly of the flagellar sheath, such as the ability to form nonbilayer structures [72,73,74] and decreasing lateral interactions within the monolayer leaflet, which lowers the energy needed to stretch membranes [75]. Localization of specific proteins to the cell pole may also contribute to formation of the flagellar sheath. Cardiolipin interacts strongly with many proteins [76] and, in some cases, cardiolipin is required for recruitment of specific proteins to the cell pole [43,77,78,79]. Additional mechanisms for localizing specific proteins to the cell pole that do not involve cardiolipin exist in rod-shaped bacteria [80], and such mechanisms could also facilitate flagellar sheath biosynthesis.

An important matter regarding flagellar sheath biogenesis is ascertaining the degree to which it is coupled to assembly of the flagellar filament. Richardson and co-workers generated non-motile mutants of *V. cholerae* following transposon mutagenesis, and identified five mutants that produced sheath-like structures that lacked the flagellar core [81]. In the coreless sheath mutants, a sheath-like structure was observed in about half the cells, and in contrast to the wild-type flagellum, the sheath-like structures were located almost always (>99%) at non-polar sites [81]. The coreless sheaths were elongated like normal sheaths, but in contrast to normal sheaths, the diameters of the coreless sheaths were irregular. These findings suggest that sheath biogenesis and flagellar assembly in *V. cholerae* can be uncoupled. This uncoupling, however, may be strain specific as the researchers were only able to isolate coreless sheath mutants from the classical strain of *V. cholerae*, and not the El Tor strain [81]. Unfortunately, the genes that were disrupted in the coreless sheath mutants were never identified, which would have provided clues that might explain the molecular basis for the unusual phenotype of these mutants. Ferooz and Letesson reported that in mutants of *B. melitensis* where *fliF* (encodes MS-ring protein), *flgE* (encodes hook protein), *fliC* (encodes flagellin) or *ftcR* (encodes flagellar gene master regulator) were deleted, coreless sheaths could be observed on some of the cells [7]. These findings are intriguing since the MS-ring is one of the earliest flagellar structures to be assembled, which suggests sheath biogenesis in *B melitensis* can initiate and proceed in the absence of any flagellar structure.

**Figure 2:**
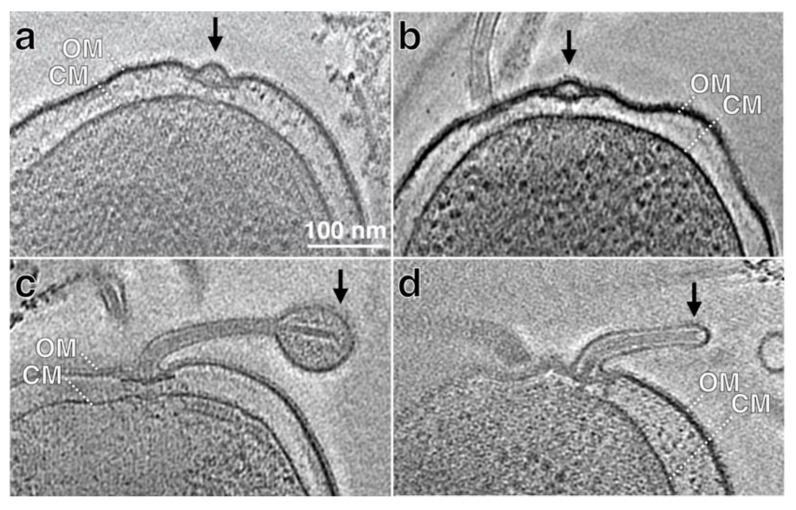
Cryo-ET reconstructions of intact cells show early stages of flagellar assembly and sheath formation. (**a**,**b**) Two representative sections from cryo-ET reconstructions of *H. pylori* cells show flagellar basal bodies without hook and filament. (**c**,**d**) Two representative sections from cryo-ET reconstructions of *H. pylori* cells show short flagellum. The outer (OM) and cytoplasmic membranes (CM) are indicated.

In contrast to the studies with *V. cholerae* and *B. melitensis* flagellar mutants [7,81], studies with wild-type *H. pylori* suggest assembly of the flagellum and sheath biogenesis are tightly coupled. In a high-throughput cryo-ET approach, Qin and co-workers visualized over 300 *H. pylori* flagella, which allowed them to image intermediate structures during flagellum assembly [32]. Figure 2 shows the intermediate structures of the flagellar sheath that are observed during flagellum assembly (author’s data). Based on the series of *H. pylori* flagellum assembly intermediates, the growing rod assembly seems to push against the outer membrane and deform it. As the hook is assembled and grows, the outer membrane is deformed further and eventually forms a bubble that surrounds the hook. During filament assembly, the flagellar sheath and filament appear to elongate simultaneously, and the bulb-like structure seen in the mature flagellum is present in the nascent flagellum [32].

**Figure 3:**
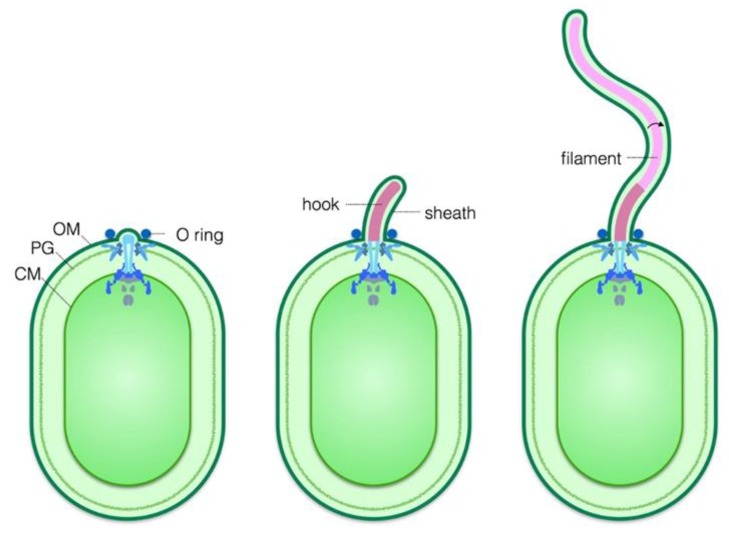
A schematic of assembly and sheath formation of a sheathed flagellum of *Vibrio alginolyticus.* The O-ring is assembled on the exterior side of the outer membrane at the point where the nascent flagellar sheath emerges. As the hook and filament are assembled, the flagellar sheath extends to encase these structures. The O-ring remains positioned at the base of the flagellar sheath where it stabilizes or induces a sharp bend in the outer membrane as it transitions into the flagellar sheath. The outer (OM), cytoplasmic membranes (CM), and peptidoglycan (PG) are indicated.

Virtually nothing is known about proteins that have roles in flagellar sheath biogenesis. The only studies that have shed any light on proteins with potential roles in sheath biosynthesis have been done with *V. alginolyticus*. In a cryo-ET analysis of the *V. alginolyticus* sheathed flagellum, Zhu and co-workers observed a ring-like structure associated with the base of the flagellar sheath [33]. The structure, designated as the O-ring, was located on the exterior side of the outer membrane, which displayed a striking 90° bend at the site of the O-ring [33]. The location of the O-ring and apparent deformation in the outer membrane that it elicits suggests a critical role for the O-ring in formation or function of the flagellar sheath. Figure 3 presents a model for assembly of the flagellum and flagellar sheath in *V. alginolyticus*. The genes encoding the O-ring protein(s) have yet to be identified, which has prevented researchers from confirming a role for the O-ring in flagellar sheath biogenesis. Structures that are analogous to the O-ring have not been identified in any other bacteria with sheathed flagella, indicating that any role for the O-ring in flagellar sheath biogenesis in *V. alginolyticus* is not universal among bacteria that possess flagellar sheaths.

The flagellar motors of many bacteria have embellishments that are absent in the archetypical flagellar motors of *E. coli* and *S. enterica* serovar Typhimurium [30,31,32,33,82]. One such embellishment is the H-ring, which is closely associated with the L-ring/P-ring complex and in close proximity to the outer membrane in *Vibrio* species [30,33]. The H-ring is located on the periplasmic side of the outer membrane, and the proteins that comprise the H-ring (FlgO and FlgT) have been identified [30,83,84]. Zhu and co-workers demonstrated that deletion of *flgO* or *flgT* in *V. alginolyticus* disrupted formation of the H-ring and resulted in a many of the flagella being located in the periplasm [84]. About 80% of the flagella in the *flgT* mutant were located in the periplasm, compared with about 10% of the flagella in the *flgO* mutant and none of the flagella in the parental strain having a periplasmic location [84]. Some of the filaments of the periplasmic flagella protruded through the outer membrane at sites that were far from the cell pole. Some of the protruding filaments were encased in a flagellar sheath, while others lacked a sheath [84]. Taken together, these observations suggest the H-ring assists the flagellum in penetrating the outer membrane and forming the flagellar sheath.

## 6. Proposed Functions for Flagellar Sheaths

A variety of functions have been proposed for bacterial flagellar sheaths, however, the lack of sheath-less mutants for any bacterial species makes it difficult to confirm proposed functions for the flagellar sheath. The flagellar sheath may have multiple functions within a given bacterial species, and functions of the sheath may vary between species. For *H. pylori*, one of the original proposed functions for the flagellar sheath was to protect the filament subunits from dissociation in the presence of gastric acid. The *H. pylori* flagellar sheath has also been proposed to be involved in adherence. In support of the proposed role of the *H. pylori* flagellar sheath in adherence, the putative adhesion HpaA is reported to be located in the flagellar sheath. While HpaA was originally described as a sialic acid binding adhesion [53,85], supporting evidence for this activity is still lacking [54,55], and so it is unclear if HpaA does indeed have a role in adherence. Nevertheless, HpaA is required for colonization of the mouse model [86].

Another proposed function for the flagellar sheath is escaping detection of the flagellins by the host innate immune system. Toll-like receptor 5 (TLR5) is a surface exposed host receptor that recognizes flagellin [87]. Binding of flagellin to TLR5 stimulates proinflammatory cytokine production, which induces an inflammatory response that can lead to active clearance of the invading bacterium and an enhancement of the adaptive immune response [88]. Yoon and Mekalanos demonstrated that compared to the unsheathed flagella of *Salmonella enterica* serovar Typhimurium, the sheathed flagella of *V. cholerae* were significantly reduced in their relative potency to trigger the host innate response [89]. The *V. cholerae* flagellins and *S. enterica* serovar Typhimurium flagellin were similar in their potencies to trigger the host innate response, indicating that the *V. cholerae* flagellar sheath is effective in hiding immunogenic flagellins [89]. TLR5 recognizes a highly conserved region of flagellin that is required for flagellum assembly in members of the Gammaproteobacteria [90]. Flagellins of *H. pylori* and other members of the Epsilonproteobacteria lack the conserved region that interacts with TLR5 [91], which suggests the flagellar sheath does not play a major role in avoiding triggering the host innate immune response by flagellin in these bacteria.

The flagellins of several bacterial species are glycosylated (i.e., post-translationally modified by the covalent attachment of carbohydrates to specific amino acids), and some of these bacteria possess flagellar sheaths [92,93]. Flagellar glycans have roles in a variety of processes, including flagellar filament assembly, motility, autoaggultination, adherence to and invasion of host cells, virulence, and evasion of the host innate immune system [94,95,96,97,98,99,100]. *H. pylori* flagellins FlaA and FlaB are modified with a single type of glycan, pseudaminic acid, and flagellin glycosylation is required for assembly of the flagellar filament [100]. Flagellin glycosylation in *Campylobacter jejuni,* which is closely related to *H. pylori*, is also required for filament assembly, but the glycans of the *C. jejuni* flagellins are much more heterogeneous, and include various derivatives of pseudaminic acid and a derivative of legionaminic acid [101,102]. Logan proposed that the *H. pylori* flagellar sheath prevents recognition of the flagellin glycan by the host immune system, which may have decreased the evolutionary pressure for glycan heterogeneity in this bacterium [92].

As discussed previously, rotation of the sheathed flagellum of *Vibrio* species releases OMVs, which are known to have important roles in host signaling in symbiosis and pathogenesis. In the symbiosis between *V. fischeri* and the Hawaiian bobtail squid, *Euprymna scolopes*, LPS associated with the OMVs induces apoptotic cell death within the surface epithelium of the squid light organ that is required for its normal development [63,64]. Vanhove and co-workers found that OMVs released from *V. tasmaniensis*, a facultative intracellular pathogen of oyster haemocytes, contained several virulence factors that could be delivered to host cells either extracellularly or intracellularly [66]. The presence of several flagellar proteins in the OMVs and the occurrence of membrane blebs on flagellar sheaths suggested that some of the OMVs originated from the flagellar sheath [66]. It is not known, however, if OMVs derived from the flagellar sheath contain virulence factors.

OMVs also have a potential role in innate bacterial defense, as Manning and Kuehn showed OMVs protected enterotoxigenic *E. coli* from certain outer membrane-acting stressors, such as antimicrobial peptides and T4 bacteriophage [103]. OMVs interacted with antimicrobial peptides in a dose-dependent manner; and irreversibly bound phage, as well as reduced the ability of phage to infect once attached to the OMV [103]. In a somewhat related study, Zhang and co-workers demonstrated that rotation of the polar sheathed flagellum reduced absorption of phage OWB to *V. parahaemolyticus* [104]. Mutations that prevented either the synthesis or rotation of the polar flagellum enhanced the ability of the phage to lyse the bacterium [104]. The authors of this study suggested rotation of the sheathed flagellum of *V. parahaemolyticus* protects the bacterium from phage by releasing OMVs that bound the phage [104]. Alternatively, rotation of the polar flagellum is a mechanosensory mechanism that regulates gene expression [105], and mutations that prevent rotation of the polar flagellum may alter the cell surface to enhance phage absorption.

Another potential function of flagellar sheaths is to protect bacteria from flagellotropic phages, a group of phages that use the flagellar filament as a host receptor for attachment. The infection mechanism of flagellotropic phages is poorly understood, but flagellar rotation is required for infection and is thought to facilitate translocation of the phage along the filament to the cell surface [106]. It is possible flagellar sheaths evolved as a mechanism to hide the flagellar filament from flagellotropic phages. Consistent with this hypothesis, we are unaware of any reports on flagellotropic phages of bacteria that possess flagellar sheaths; although flagellotropic phages of bacterial species closely related to bacteria that have flagellar sheaths have been reported, such as phage F342 of *C. jejuni* [107].

## 7. Conclusions and Future Directions

The 1983 review of bacterial flagellar sheaths by Sjoblad and colleagues begins with the statement, “Although bacterial flagellar sheaths were observed over 30 years ago, they may still be characterized as structures in search of a function” [3]. Some of the assumed roles for flagellar sheaths in 1983, such as adherence, are still considered as possible roles for flagellar sheaths today. And although additional roles have been postulated for bacterial flagellar sheaths over the last 37 years, limitations in our knowledge of flagellar sheath biosynthesis and the lack of mutants that synthesize sheath-less flagella thwart efforts to confirm proposed functions for flagellar sheaths.

The limited number of studies that have examined the composition of flagellar sheaths have indicted the sheath is both similar to and different from the outer membrane. Differences in the LPS composition of flagellar sheaths and the outer membrane indicated by some studies [29,38] require an unknown mechanism to segregate specific LPS species within what appears to otherwise be a contiguous membrane. Mechanisms for localizing specific proteins to the sheath are easier to envision. For example, bacterial proteins are localized to the cell pole through a diffusion-capture mechanism in which proteins are inserted into the membrane where they can diffuse until encountering a geometrical cue (e.g., membrane curvature) or biochemical cue (e.g., specific phospholipids or other proteins already localized to the site) [80]. Given the unique physiochemical properties of flagellar sheaths (e.g., shape, phospholipid composition), such a diffusion-capture mechanism is likely to be responsible for the localization of proteins to the flagellar sheath. Studies in *V. cholerae* and *H. pylori* have identified proteins that appear to localize to the flagellar sheath [51,52,55,57], and future studies in these bacteria, as well as other bacterial species, will most certainly lead to the identification of additional flagellar sheath proteins. Dissecting the lipid and protein composition of bacterial flagellar sheaths is critical for understanding the function and biogenesis of these unique structures.

One of the most fascinating areas for future investigations into flagellar sheaths is understanding how these structures are assembled. Making headway in understanding the molecular mechanisms that control flagellar sheath biogenesis will require a combination of genetic, biochemical, and structural approaches. High-throughput cryo-ET studies, like that done by Qin and co-workers with *H. pylori* [32], will need to be done with other bacterial species. Identifying and disrupting genes that encode structural features intimately associated with flagellar sheaths, such as the O-ring of *V. alginolyticus*, will be required to ascertain the roles these genes play in flagellar sheath biosynthesis. Creative genetic screens will be needed to identify genes that are required for flagellar sheath biosynthesis and, hopefully, lead to the generation of mutant strains that produce sheath-less flagella, which can be used to test the requirement of the flagellar sheath in host colonization and pathogenesis. Biochemical studies will be needed to examine the composition of flagellar sheaths, as well as confirm the predicted activities of the products of candidate genes for sheath biosynthesis identified through genetic and genomic approaches.
